# Pearls and Pitfalls of Introducing Ketogenic Diet in Adult Status Epilepticus: A Practical Guide for the Intensivist

**DOI:** 10.3390/jcm10040881

**Published:** 2021-02-22

**Authors:** Jason B. Katz, Kent Owusu, Ilisa Nussbaum, Rachel Beekman, Nicholas A. DeFilippo, Emily J. Gilmore, Lawrence J. Hirsch, Mackenzie C. Cervenka, Carolina B. Maciel

**Affiliations:** 1 Department of Neurology, Neurocritical Care Division, UF Health-Shands Hospital, University of Florida, Gainesville, FL 32611, USA; jasonbkatz@ufl.edu; 2 Department of Neurology, Yale New Haven Hospital, Yale School of Medicine, New Haven, CT 06520, USA; kent.owusu@ynhh.org (K.O.); ilisa.nussbaum@ynhh.org (I.N.); rachel.beekman@yale.edu (R.B.); emily.gilmore@yale.edu (E.J.G.); lawrence.hirsch@yale.edu (L.J.H.); 3 Care Signature, Yale New Haven Health, New Haven, CT 06510, USA; 4 Department of Pharmacy Services, Yale New Haven Hospital, New Haven, CT 06510, USA; nicholas.defilippo@uconn.edu; 5 School of Pharmacy, University of Connecticut, Storrs, CT 06269, USA; 6 Department of Neurology, Epilepsy Division, Johns Hopkins Hospital, Johns Hopkins University School of Medicine, Baltimore, MD 21287, USA; mcerven1@jhmi.edu

**Keywords:** ketogenic diet, status epilepticus, new onset refractory status epilepticus, seizures, critical care, ketosis

## Abstract

**Background:** Status epilepticus (SE) carries an exceedingly high mortality and morbidity, often warranting an aggressive therapeutic approach. Recently, the implementation of a ketogenic diet (KD) in adults with refractory and super-refractory SE has been shown to be feasible and effective. **Methods:** We describe our experience, including the challenges of achieving and maintaining ketosis, in an adult with new onset refractory status epilepticus (NORSE). **Case Vignette:** A previously healthy 29-year-old woman was admitted with cryptogenic NORSE following a febrile illness; course was complicated by prolonged super-refractory SE. A comprehensive work-up was notable only for mild cerebral spinal fluid (CSF) pleocytosis, elevated nonspecific serum inflammatory markers, and edematous hippocampi with associated diffusion restriction on magnetic resonance imaging (MRI). Repeat CSF testing was normal and serial MRIs demonstrated resolution of edema and diffusion restriction with progressive hippocampal and diffuse atrophy. She required prolonged therapeutic coma with high anesthetic infusion rates, 16 antiseizure drug (ASD) trials, empiric immunosuppression and partial bilateral oophorectomy. Enteral ketogenic formula was started on hospital day 28. However, sustained beta-hydroxybutyrate levels >2 mmol/L were only achieved 37 days later following a comprehensive adjustment of the care plan. KD was challenging to maintain in the intensive care unit (ICU) and was discontinued due to poor nutritional state and pressure ulcers. KD was restarted again in a non-ICU unit facilitating ASD tapering without re-emergence of SE. **Discussion:** There are inconspicuous carbohydrates in commonly administered medications for SE including antibiotics, electrolyte repletion formulations, different preparations of the same drug (i.e., parenteral, tablet, or suspension) and even solutions used for oral care―all challenging the use of KD in the hospitalized patient. Tailoring comprehensive care and awareness of possible complications of KD are important for the successful implementation and maintenance of ketosis.

## 1. Introduction

Status epilepticus (SE) carries an exceedingly high mortality and morbidity, often warranting an aggressive therapeutic approach. Recently, the implementation of ketogenic diet (KD) in adults with refractory and super-refractory SE has been shown to be feasible and potentially effective [1,2,3,4,5]. Most often used in childhood epilepsies, KD has emerged as a potential adjunctive treatment for pediatric SE [6,7]. We describe our experience with an adult with new onset refractory status epilepticus (NORSE) focusing on the unexpected challenge of achieving and maintaining ketosis. Practical advice, and a comprehensive review of factors potentially jeopardizing ketosis commonly encountered in the critical care setting and alternatives are provided.

## 2. Presentation

*A previously healthy 29-year-old woman was admitted to another institution with new onset refractory status epilepticus (NORSE) following a febrile illness with a course complicated by prolonged super-refractory SE. Three days prior to presentation she developed fever, headache, emesis and fatigue in the setting of being in contact with her child with an upper respiratory tract infection. On the morning of admission, her friend attempted to awaken her for work and found her unresponsive and convulsing. In the emergency department, she was lethargic and mumbling incoherently. During her initial evaluation she had a witnessed 45-s bilateral tonic-clonic seizure that was aborted with 2 mg lorazepam intravenously. Head computed tomography was unremarkable and initial cerebrospinal fluid (CSF) analysis showed a mononuclear pleocytosis (2 RBC, 41 nucleated cells (57% mononuclear cells), glucose 93, protein 54)). A one-hour electroencephalogram (EEG) showed diffuse delta activity admixed with sleep spindles and K complexes without epileptiform discharges. She was monitored in the step-down unit and treated with levetiracetam and acyclovir. On hospital day two, she was somnolent but arousable to voice; she was able to follow simple midline commands, state her name and the current president, but was disoriented to time. She was noted to have twitching of her face, but no EEG was done at that time. By hospital day three, she began experiencing brief convulsive seizures which were aborted with intravenous lorazepam and always associated with recovery of consciousness. At that point, phenytoin (1 g loading dose, maintenance at 100 mg q8h) was added to her antiseizure drug (ASD) regimen. On hospital day four, she had multiple convulsive seizures without return to baseline, complicated by acute hypoxic respiratory failure requiring intubation. She was transferred to the intensive care unit (ICU) where she was started on propofol, and valproic acid (20 mg/kg loading dose, maintenance at 750 mg Q8H) was added. Routine EEG captured multiple discrete right frontal and centrotemporal onset seizures correlating with episodes of face twitching. She was started on pentobarbital infusion (5 mg/kg bolus, maintenance at 1 mg/kg per hour) and transferred to our center for continuous EEG monitoring.*

## 3. Question: How Is Prolonged Seizure Activity Classified and What Are Potential Etiologies to Be Considered?

According to the most recent classification set by The International League Against Epilepsy, SE is a “condition resulting either from the failure of the mechanisms responsible for seizure termination or from the initiation of mechanisms, which lead to abnormally, prolonged seizures” [8]. While operational SE definitions based on time-domains vary according to seizure type, it is generally accepted that convulsive seizure activity lasting either greater than 5 min continuously, or two or more seizures during which the individual does not recover to baseline between seizures, represents SE [8]. Inhibitory gamma-aminobutyric acid (GABA) neurons located in the pars reticulata of the substantia nigra are key in seizure termination [9]. During status epilepticus, marked alteration of GABA metabolism occurs in this region and results in disinhibition of excitatory pathways: GABA synthesis slows down [10]. GABA turnover time increases up to three-fold, [10] and GABA receptors (originally located in the surface of the cell membrane) migrate to the intracellular space within minutes of ongoing seizure activity [11,12,13]. Prompt initiation of abortive therapies is key, as the internalization of GABA receptors contributes to refractoriness to treatment.

Emergent administration of parenteral benzodiazepine (e.g., up to 0.1 mg/kg of lorazepam) is considered the first-line therapy for SE [14]. If a patient fails to respond to a benzodiazepine and a second appropriately selected and dosed ASD at adequate doses, they are in refractory status epilepticus (RSE). It has been reported that one in five RSE patients go on to develop super-refractory status epilepticus (SRSE), defined as (1) ongoing seizures lasting 24 h or more after onset of anesthetic therapy (i.e., propofol) or (2) recurrence of SE upon reduction or withdrawal of therapeutic anesthetic coma [15]. The clinical presentation of RSE in patients without overt acute or remote brain injury, prior epilepsy, or acute toxic/metabolic explanation is consistent with New Onset Refractory Status Epilepticus—NORSE [16]. Febrile Infection-Related Epilepsy Syndrome (FIRES) is a subset of NORSE, in which a febrile infectious illness precedes SE onset by 1–14 days [14,15,16]. Thus, our patient qualified as having the syndrome of FIRES as well as NORSE. Further, clinical criteria for unequivocal electroencephalographic status epilepticus in patients without known epileptic encephalopathy has been established in the Salzburg consensus: (1) repeating epileptiform discharges occurring >2.5 Hz, or (2) repeating epileptiform discharges occurring ≤2.5 Hz or rhythmic delta/theta activity >0.5 Hz plus (a) electroclinical response (improvement) following intravenous ASD challenge, (b) subtle clinical correlate associated with pattern, or (c) typical temporal and spatial evolution of pattern [17].

Determining the underlying etiology of SE may seem a daunting task. However, its importance in achieving seizure cessation cannot be underestimated. Outcome following SE is dependent on the etiology of seizures. Further, appropriately recognizing the electroclinical classification of seizures (i.e., identifying the seizure semiology and its electrographic signature) may not only help selection of therapy but also identify potential etiologies [8]. Among NORSE patients, an etiology is found in up to 50% of cases [16]. Of those with an identifiable cause, the majority (37%) had an autoimmune cause (both nonparaneoplatic and paraneoplastic), while 8% had a probable infectious cause [16]. Thus, if an autoimmune etiology is diagnosed or clinically suspected, immune modulating therapies such as high-dose corticosteroids, intravenous immunoglobulin (IVIg) or plasma exchange therapy, followed by monoclonal antibodies and/or interleukin inhibitors, should be considered early in the course.

*Our patient underwent a comprehensive work-up to determine the etiology of NORSE, as summarized on Table 1, which was notable only for mild CSF pleocytosis, elevated nonspecific inflammatory serum markers and edematous hippocampi with associated diffusion restriction on magnetic resonance imaging (MRI).*

## 4. Question: What Are the Initial Steps in the Therapeutic Algorithm for Status Epilepticus?

Benzodiazepines are the first-line treatment for SE [14,19] with slower-acting, less sedating parenteral ASDs being the second line (e.g., phenytoin, fosphenytoin, valproate and levetiracetam; and possibly lacosamide and phenobarbital). The Established Status Epilepticus Treatment Trial (ESETT) found no difference in efficacy between fosphenytoin (20 mgPE/kg), valproate (40 mg/kg) and levetiracetam (60 mg/kg) in children, adults and older adults; these ASDs were able to abort SE within an hour in nearly 50% of patients [20]. Once the second-line, or a combination of medications, fails to result in seizure cessation, continuous intravenous infusions of anesthetics (i.e., midazolam, pentobarbital, propofol, ketamine) are often recommended [14,19].

In patients with SE, anesthetic use is associated with longer hospital stay, but not in-hospital or 90-day mortality [21]. Amongst NORSE patients who receive anesthetics, the mortality is high. However, the use of anesthetics is not associated with poor outcome [16]. In 61 patients with RSE, those who underwent deep sedation (defined as either EEG showing burst suppression or isoelectric activity) had both poorer long-term prognosis and increased mortality [22]. Burst suppression on EEG is defined as intermittent alternating periods of low amplitudes (<10 uV for burst suppression; 10–20 uV for burst attenuation) interrupting a background, which may consist of waves of varying frequencies. Earlier attainment of burst suppression may allow for a more rapid anesthetic wean [23], and it is important to frequently monitor the EEG and titrate anesthetic dose as appropriate. The goal of anesthetic use in RSE is the resolution of epileptiform activity in order to avoid physiologic effects while the underlying cause is identified and treated [24,25].

*In our case, 16 antiseizure drug trials in various combinations and high anesthetic infusion rates were attempted. RSE persisted despite 160 mg/h (2.5 mg/kg/h) of midazolam prompting the initiation of ketamine. Despite improved seizure burden following ketamine bolus (1.5 mg/kg), reemergence of SE occurred despite up-titration of ketamine to our maximum infusion rate (7.5 mg/kg/h). Her EEG responded to propofol and pentobarbital with long periods of suppression, although her background remained with abundant generalized periodic discharges (GPD) at 2.5–3 Hz, qualifying as ongoing electrographic SE.* [17,26] *Burst suppression was eventually achieved with pentobarbital at 3 mg/kg/h and propofol at 40 mcg/kg/min. However, due to re-emergence of 2 Hz GPDs along with breakthrough seizures on attempted wean, she remained in a medically induced coma for over three months.*

## 5. Question: What Are Potential Rescue Therapeutic Approaches to the Management of Super-Refractory Status Epilepticus?

Refractory and super-refractory SE and their complications are associated with significant morbidity including death, neuronal damage and systemic complications like cardiomyopathy, ischemic bowel, pulmonary edema and renal failure [27]. This highlights the necessity for a prompt and aggressive treatment approach. In the setting of treatment failure, alternative treatment options include inhaled anesthetics, magnesium infusion, pyridoxine, hypothermia, electrical and magnetic stimulation, additional immunotherapy, enteral ASDs and the KD [28].

*Given a high suspicion for an autoimmune process, our patient was treated with intravenous methylprednisolone (1 g daily for 5 days), IVIg, plasma exchange and cyclophosphamide. Serial MRIs demonstrated resolution of edema and diffusion restriction with gradually progressive atrophy, predominantly in the hippocampi, and repeat CSF analysis was normal. She underwent empiric bilateral partial oophorectomy for an echogenic focus in her left ovary and concern for possible occult microteratoma, possibly secondary to N-methyl-D-Aspartate (NMDA) encephalitis (NMDA CSF < 1:1 and serum < 1:10) [29].*

## 6. Ketogenic Diet

Ketosis is commonly defined as sustained beta-hydroxybutyrate levels > 2 mmol/L [30] or a urinary acetoacetate level of >40 mg/dL [31]. There is evidence supporting the use of KD in children with autoimmune epilepsies, symptomatic epilepsy syndromes, pediatric refractory and super-refractory SE [6,32]. In a study of 10 children (age six months—16 years old) with refractory focal SE, initiation of a KD resulted in lower seizure burden (50% reduction in seizures for 70% of the cohort) and resolution of seizures in 20% [6]. In the minority of patients with less than 50% seizure reduction (*n* = 3), severe adverse events (pancreatitis or severe vomiting and hypoglycemia) prompted KD discontinuation. In another study of 12 children with fever induced refractory epileptic encephalopathy, KD was able to stop seizures within two days following ketonuria [32]. Nevertheless, the side effects of KD limit its widespread use, and successful ketosis must be attained for seizure control.

More recently, KD has been evaluated in adult patients; a systematic review of 38 adult patients with RSE or SRSE demonstrated that 82% were able to achieve SE cessation with KD [33]. There are several complex mechanisms for the efficacious effect of KD on reducing seizure activity, which result from reduction in glucose intake, ketone body production and alteration of the gut microbiome. The metabolic changes induced by KD alter the balance of excitatory and inhibitory neurotransmitters, lead to reductions in oxidative stress and systemic as well as neuroinflammation, and have further long-term effects on gene expression [3,34].

*We sought KD as a rescue therapy after conventional treatments had failed.*

## 7. Question: What Are Some Factors Should the Clinician Consider When Selecting and Initiating KD for Adults with SE?

### Initiating KD Safely

Determining the optimal patient for whom to implement KD requires a comprehensive evaluation of the patient’s past medical history, comorbidities and current clinical status. As with all treatment strategies, particularly in the ICU setting, a thoughtful risk-benefit analysis is warranted. Inborn errors of metabolism are a contraindication to KD [35,36]. However, these conditions most often present in early childhood, and rarely in adults, so screening is not routinely obtained prior to KD initiation in adults [1,2]. Other contraindications of KD include unstable metabolic (mitochondrial enzyme deficiencies) conditions, liver failure, acute pancreatitis, pregnancy and an inability to tolerate enteral feeds [5]. Protocols typically avoid starting KD within 24 h of propofol infusions to avoid possibly fatal propofol infusion syndrome, characterized by metabolic acidosis, lipemia, rhabdomyolysis and myocardial failure [37].

## 8. Question: Should You Fast the Patient to Achieve Ketosis Quickly? If So, How Long and What Are Potential Consequences? If You Decide Not to Fast, Can Ketosis Still Be Achieved?

### Variations in KD Protocols

Historically, KD implementation in the setting of childhood epilepsy included an initial fasting period ranging anywhere from 12 [7] to 48 [36,38] hours or more. Once satisfactory ketosis is achieved, ketogenic formulations or meals (typically 4:1 g of fat: carbohydrate + protein ratio) can then be titrated as tolerated until full caloric requirements are met. To avoid potential complications of a fasting period (e.g., dehydration, hypoglycemia), Kim et al. began KD without initial fasting and found equivalency in time to ketosis and seizure reduction in 41 children with intractable epilepsy compared to a retrospective control population of 83 children who fasted prior to KD initiation [39]. While rates of hypoglycemia were similar when compared to controls, there were reduced rates of dehydration and reduced length of hospital stay.

An alternative, yet equally efficacious approach for childhood epilepsy, does not involve initial fasting or limiting caloric intake. This protocol differs from others in the fact that there is a gradual increase from 1:1 to 2:1 until the goal 4:1 ratio is reached [38]. This gradual induction and establishment of ketosis in children diagnosed with intractable epilepsy showed an equal reduction in seizure activity yet decreased weight loss and episodes of hypoglycemia, acidosis and dehydration. Nevertheless, since time is a major factor in terms of avoiding neurologic and systemic consequences of SE, a more aggressive approach to KD initiation (i.e., fasting and/or more rapidly advancing to full calories as tolerated) may be warranted in this setting.

Individual patient characteristics including age, illness severity, duration of anesthetic use prior to diet initiation resulting in reduction in gastrointestinal motility, and diet complications, may not allow the luxury of initiating a preferred protocol with certain ketogenic ratio or at a faster rate. This was evident in Cobo’s pediatric SRSE study in which ratios were started as low as 0.75:1 in some instances, and ratios never exceeding 2:1 in some cases [7]. The need for higher protein intake (often in cases of poor wound healing, malnutrition and/or low basal resting energy expenditure) challenges the use of higher fat:protein + carbohydrate ratios, although this is more of a concern with chronic KD use rather than in the acute setting of RSE and SRSE. A possible way to maximize ketosis when using lower ratios (thus, higher protein intake) is the addition of medium-chain triglyceride oils as they yield greater amounts of ketones/kcal of energy than longer chain varieties [36].

**Figure 1:**
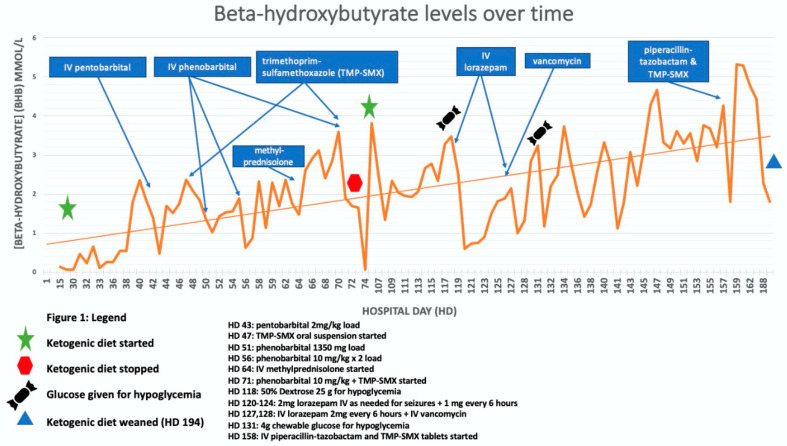
Drug Interference with achievement of ketosis. Seizure Activity: On HD 14–16, seizure burden was 90% nonconvulsive status epilepticus (NCSE) and decreased to 10–15% by hospital day (HD) 23–24. From HD 30–39, % ictal ranged from 5–20% with HD 40 showing <1% ictal. % Ictal increased briefly during HD 41–47 with an average of 15% ictal but decreased to <1% by HD 48/49. % Ictal remained in the 10–20% range until HD 57 with % ictal < 1. From HD 58–72 % ictal ranged from 5–20% until % ictal <5 by HD 72.

*We used these principles, most frequently used in the setting of childhood epilepsy, to initiate KD for our NORSE patient with the goal of achieving ketosis quickly. Our patient was initially started on KD on hospital day 28 (HD 28) with a goal of 5:1 ratio (KetoCal^®^ 4:1 at 55 mL/h plus 33 mL medium-chain triglycerides (MCT) oil to balance carbohydrate intake from medications, documented as 51 g daily on HD 30). At this time, supplemental protein *via* PROsource^®^ was discontinued to assist with achieving ketosis. On HD 35, beta-hydroxybutyrate (BHB) levels continued to show inadequate ketosis [Figure 1] prompting the increase to 6:1 with additional MCT Oil. Through HD 65, beta-hydroxybutyrate continued to fluctuate below the 2.0 goal. On HD 71, beta-hydroxybutyrate again dropped with the only documented potential carbohydrate source (at that time) being a milk and molasses enema administered by a care team to alleviate constipation. The decision was made to return to a higher carbohydrate-containing formula and refocus nutrition goals on wound healing. At this time, our patient was identified as meeting the criteria for severe malnutrition based on weight loss of >7.5% in three months and limited energy intake for greater than or equal to five days* [40].

## 9. Question: What Factors Can Impede the Success of Achieving Ketosis, and thus Jeopardize the Utility of KD?

*Despite initiating KD with complete enteral feeds on hospital day 28, our patient was only able to reach ketosis a significant 37 days later (Figure 1).* After a comprehensive assessment of the care plan, the culprit was found: inconspicuous carbohydrate-containing medications, infusions and oral-care solutions routinely given in the setting of a neurological ICU (Table 2).

## 10. Hidden Carbohydrates Can Hinder Achievement of Ketosis

In our experience, the most likely medication-induced barriers to ketosis include sedatives, antiseizure drugs and antibiotics. Benzodiazepines are not created equally when it comes to hidden/nonobvious carbohydrate content. Diazepam (Valium^®^ 5 mg/mL) and Lorazepam (Ativan^®^ 2 mg/mL) have been shown to have 40% and 80% propylene glycol content, respectively, which equates to an overall carbohydrate content of 0.4 g/mL and 0.8 g/mL [42]. Intake of this carbohydrate content in patients with status epilepticus on KD may hinder achieving ketosis, but can also precipitate propylene glycol toxicity and associated anion-gap, metabolic acidosis [44].

While the carbohydrate load of each individual dose may be inconsequential, the cumulative dose given to patients in extended hospital stays may be significant. For example, initial administrations of injectable lorazepam, 2 mg every 6 h yields approximately 3 g of carbohydrate per day [44]. *For context, this patient’s energy assessment used an ideal body weight of 64.5 kg, with daily caloric requirements calculated to total 2260 kcal/day (35 kcal/kg). Using KD at a 4:1 ratio (a common target for KD), the macronutrient breakdown is 226 g fat, 51.6 g protein, and 4.9 g carbohydrates.* Thus, lorazepam would have contributed over half of the allotted daily carbohydrate load. *Figure 1 shows several instances where lorazepam administration was associated with significant troughs in beta-hydroxybutyrate levels.* When benzodiazepine infusions are warranted for refractory cases, our recommendation would be to consider midazolam as an alternative; midazolam does not have propylene glycol in its formulation, resulting in lower rates of anion-gap metabolic acidosis [45]. Regarding enteral administration of benzodiazepines, it is worth noting that clobazam (Onfi ^®^) and clonazepam (Klonopin^®^) contain 105 mg and 143.5 mg of carbohydrates (i.e., lactose, starch) per 10 mg/0.5 mg tablet, respectively [Table 2]. When selecting benzodiazepines for treatment of SE in patients treated with KD, it is important to evaluate the carbohydrate content for the selected benzodiazepine and the administration method. Generally, solutions and suspensions should be avoided due to high carbohydrate containing excipients.

Another class of widely used sedatives, often with the potential to inadvertently hinder beta-hydroxybutyrate levels, are the barbiturates, particularly pentobarbital (Nembutal^®^ 50 mg/mL) and phenobarbital (Phenobarb^®^ 130 mg/mL). Barbiturates are commonly used in RSE. However, they have a high propylene glycol content (pentobarbital 414 mg/50 mg vial, phenobarbital 702 mg/130 mg vial [42] amounting to an overall carbohydrate content of 2.9 g/h when pentobarbital is infused at a rate of 5 mg/kg/h for a 70 kg patient) [Table 2]. We suggest that ketamine (and midazolam, as discussed above) be considered as an alternative to pentobarbital in patients with status epilepticus on KD, given their lack of propylene glycol [46], This same concept can be applied for phenytoin (Dilantin^®^ 50 mg/mL), which contains 40% propylene glycol [42] amounting to a carbohydrate content of 414 mg (plus an additional 79 mg of alcohol). *These principles can be observed in Figure 1 where beta-hydroxybutyrate levels drop on HD 52, corresponding with a phenobarbital load (a disruption in feeds also occurred to administer phenytoin by mouth).* We recommend that fosphenytoin be used instead to avoid propylene glycol excipients (Table 2). *Similarly, on HD 56, beta-hydroxybutyrate dropped to 0.63 mmol/L with a medication review showing intravenous phenobarbital administered overnight.* Lastly, another note of caution regarding anesthetic infusions: propofol contains 1.1 kcal/mL (mostly from fats), which provides approximately 528 kcal per day (presuming 20 mL/h). While this increased caloric load may aid in ketosis, its 450 mg of glycerol per 20 mL vial may hinder ketosis (Table 2).

## 11. Noncarbohydrate Related Hindrance of Ketosis

Among children with refractory epilepsy, concomitant lamotrigine use decreases KD’s efficacy in seizure reduction [47]. This may be explained by ketosis increasing the metabolism/inactivation of lamotrigine via glucosyltransferases, which ultimately results in increased glutamate release. Lastly, one must not forget that while medication use can influence KD, the inverse is also true. While most serum concentrations of ASD were not found to significantly change upon KD use, valproic acid levels have been shown to decrease [48]. Therefore, it is recommended to monitor valproic acid while using KD therapy.

## 12. Question: Aside from Sedative Agents, What Other Widely Used Agents in the Neurological ICU Can Hinder Ketosis?

### 12.1. Antimicrobials and Respective Diluents as Source of Carbohydrates

Various antibiotics that are used frequently in the neurological ICU [49,50] can hinder ketosis. Intravenous trimethoprim-sulfamethoxazole (TMP-SMX, Bactrim^®^) requires reconstitution with dextrose 5% water and, similarly, vancomycin is often diluted in dextrose 5% water prior to intravenous administration (Table 2). *Figure 1 (line graph) shows several instances in which administration of TMP-SMX and vancomycin were associated with troughs in beta-hydroxybutyrate levels.*

### 12.2. Non “Medications” Contain Hidden Carbohydrates

Hidden carbohydrates are found in oral care solutions such as chlorhexidine, dietary supplements and fiber. Chlorhexidine 0.12% oral solution (Peridex^TM^), reported to be superior to toothbrushing at reducing early ventilator-associated pneumonia [51], contains glycerin and ethyl alcohol (see Appendix A), both carbohydrate-containing substances that can affect KD [52]. Oral fiber supplements, such as psyllium, have a significant carbohydrate load (e.g., 9 g/tablespoon) (Table 2). However, as fiber has a lower glycemic index compared to other carbohydrate-containing sources in the ICU setting, it may still be used in some instances to counteract constipation.

### 12.3. Comprehensive Approach to Implementing KD in Adult SE

Adopting a systematic approach is key for the successful implementation and maintenance of a ketotic state. A checklist is provided in Table 3 with a summary of the suggested steps for successful KD initiation [4].

## 13. Question: What Laboratory Values Should the Intensivist Pay Particular Attention to When Using KD?

Baseline assessment of certain serum (lipid panel, complete metabolic panel, complete blood count, amylase, lipase, Vitamin D, and free and total carnitine) [41] and anthropometric (weight, height) parameters [5,33,53] are imperative to effectively see how values trend overtime with KD. This objective data collected longitudinally allows monitoring of metabolic and systemic side effects of KD to allow for cessation if need-be.

After deciding that KD is appropriate, the next step is to carefully review all standing orders in the patient’s chart to identify potential sources of hidden carbohydrates and replace with alternatives. Guidance by the dietary/nutrition team as well as an ICU pharmacist are recommended to improve success with achieving and maintaining ketosis. If possible, a nutritionist with knowledge and experience with managing a KD is preferred, which may necessitate involving the pediatric nutrition team.

In the critical care setting, nutrition is often dictated by the critical care team via administration of formula tube feeding, and patient compliance is not a key factor for achieving ketosis. Route of administration does impact ketosis as suspension or elixir medications, [53] the preferred formulations via percutaneous gastrostomies (PEG) or other enteral routes, must be replaced by alternative formulations with lower carbohydrate content [7]. Additionally, dextrose-free diluents [52] must be used whenever able in intravenous drug formulations. Unavoidable carbohydrates can be balanced with a calculated dose of fat in the form of MCT oil or a commercially available emulsified oil (provided that this addition coincides with caloric and macronutrient percentages discussed later). Additionally, this puts the patient at an increased risk for gastrointestinal complications such as steatorrhea, emesis and reflux.

## 14. Ketosis Maintenance and Surveillance

Inducing ketosis is only the first step in tackling SE. While a ketotic state is commonly defined as beta-hydroxybutyrate >2 mmol/L [30], similarly to antiseizure drugs, some individuals require higher level of ketosis (or antiseizure drugs) to achieve optimal seizure control. Thus, individual thresholds for optimal ketotic state may vary. *In the presented case, the best therapeutic effect was noted with beta-hydroxybutyrate >3.5.* Maintaining ketosis above a certain therapeutic level proves to be difficult, particularly when higher targets are required such as >3 mmol/L beta-hydroxybutyrate [7]. For example, Cobo’s case series illustrated that children with SRSE can have sudden, unexplained drops in beta-hydroxybutyrate levels.

*After KD was stopped on HD 73 due to poor nutritional status, KD was reinitiated on HD 106 as the patient was no longer on bolus medications with high carbohydrate content. Ketosis was achieved rapidly over two days, with a beta-hydroxybutyrate of 2.34 mmol/L target once the goal of 6:1 ratio 18 kcal/kg was reached. The rapid achievement of ketotic state likely resulted from several days of fasting prior to its initiation in the setting of percutaneous gastrostomy placement and enterocutaneous fistula repair. The patient remained in ketosis, with beta-hydroxybutyrate levels fluctuating between 1.93–5.32 mmol/L, during which time seizures were best controlled when beta-hydroxybutyrate levels were >3.5 mmol/L.*

Constant evaluation and re-evaluation of patient intake, including intravenous fluids, must be conducted. It is imperative to remove all common exogenous carbohydrates including glycerin, maltodextrin, propylene glycol, dextrose, fructose, glucose, lactose, sucrose, corn syrup, sugar alcohols and starches [52]. Appropriate alternatives include normal saline, balanced crystalloid or lactated Ringer’s solution.

## 15. Question: What Natural Physiologic Mechanisms Must Be Accounted for When Attempting to Achieve/Maintain Ketosis?

In addition to exogenous carbohydrate management, the intensivist must not overlook endogenous perturbations of glucose homeostasis. Commonly measured anywhere from every four [54] to eight hours after initiation of KD [7], fluctuations are commonly seen in the pediatric population as the clinician attempts to titrate to a glucose target level of 60–79 mg/dL [7]. While the lower limit of 40 mg/dL is referenced in the Academy of Nutrition and Dietetics Practice Paper, the clinicians at Yale New Haven Hospital use a slightly higher limit of 50 mg/dL when evaluating for KD initiation. Ketosis can be threatened by endogenous gluconeogenesis occurring during infection or injury [55]. The topic of glucose perturbations brings up the discussion of whether the use of glucocorticoids concurrently with KD hinders the diet’s efficacy. Among children with epilepsy being treated with KD, there have been reports of glucocorticoid use (even inhaled) being associated with seizure return, elevated glucose and ketosis hindrance [56]. More studies are needed to examine the relationships of KD and glucocorticoid use in the setting of SE/RSE.

## 16. KD and Supplements

### Question: What Supplements may Be Warranted when Starting a Ketogenic Diet?

The KD’s inherent shift into fatty acid beta-oxidation predisposes to metabolic acidosis, which can be further compounded if individuals are fasting to achieve ketosis [36]. For these reasons, adequate bicarbonate levels (commonly >17 mmol/L) [7] should be ensured with concomitant sodium bicarbonate [5] and/or potassium citrate supplementation [7].

Beta-oxidation in the mitochondria is reliant on the adequate transport of long-chain fatty acids across the mitochondrial membrane via carnitine [36,53]. Carnitine supplementation is recommended when levels are low (<30 μmol/L) or if the patient is symptomatic, defined by lethargy, weakness and GI symptoms, which are often difficult to assess in a comatose patient [7,36,53]. Carnitine supplementation remains controversial, as levels poorly correlate with tissue stores and symptoms of carnitine deficiency may be difficult to identify in comatose patients [53].

Lastly, it is recommended that a low carbohydrate multivitamin, calcium carbonate and Vitamin D be added [34,36] via nasogastric/gastric tube (NG/G-tube). Most commercially available ketogenic formulas have the recommended daily allowance of these substances. Children with epilepsy have hypovitaminosis D (50%) and are at risk for osteoporosis [36,53]. Phosphorous, [53] administered separately to avoid calcium chelation, is a recommended supplement for its role in bone homeostasis. Lastly, it is our recommendation to supplement either lite salt or table salt for patients that have hyponatremia despite administration of sodium containing intravenous fluids.

## 17. Termination of Ketotic Therapy

### Question: Once Anesthetics Have Been Weaned and/or Seizure Activity Has Improved, How Should KD Be Weaned?

Reasons for diet discontinuation include lack of response, development of complications and need for optimization of nutritional status. There are no clear guidelines to define a clear response to KD, as diseases and patient populations are very heterogeneous, and a clear absolute seizure cessation effect may not be seen. In some cases, allowing for anesthetic wean or antiseizure regimen simplification may be considered successful results. It is our recommendation that before considering therapeutic failure, higher ketotic levels should be pursued if the patient is able to tolerate a more aggressive titration of the KD, as patients, such as our patient, may respond to higher beta-hydroxybutyrate levels.

Like any antiseizure therapy, it is generally recommended to wean the KD diet gradually due to the historical thought that abrupt withdrawal of ketosis can precipitate recurrence of seizures or SE. Abrupt withdrawal of KD is recommended for emergencies only. Thus, gradually reducing the ratio of grams of fats:protein + carbohydrates is recommended (i.e., 4:1 to 3:1 to 2:1) [36]. Despite there being a common notion that overzealous weaning of KD can precipitate previously suppressed seizure activity, a study of over 183 children showed no significant difference in the incidence of seizures worsening between discontinuation/weaning rates (i.e., <1 week vs. 1–6 weeks vs. >6 weeks) [57]. However, there was an increase in seizure activity with faster weaning schedules among a particular cohort: children who had higher percentage (55–90%) of seizure reduction while on the KD. Additionally, among children who successfully stopped KD after seizure cessation, 42% of them were unable to achieve symptom improvement with either ASD or reinitiating of KD upon seizure relapse [58].

We recommend that the clinician use the KD’s treatment success as well as clinical judgement in adopting an individualized weaning schedule. *Beginning on HD 194, our patient was weaned from the KD over five days by decreasing the ketogenic formula by 20% every 24 h and replacing it with a traditional critical care formula. Supplemental MCT oil was decreased at the beginning of the weaning process. No complications arose during this transition.* Once able to tolerate oral nutrition, the patient will have the autonomy to determine whether to continue KD and contingency plans, such as offering a less strict KD therapy (modified Atkins diet, modified KD), which may be a reasonable alternative.

## 18. Anticipating and Managing Complications

### Question: What Are Some Potential Complications of KD?

Complications of KD are not uncommon and may result in discontinuation of the diet. In the pediatric literature, 30 [6] to 38% discontinuation rates [59] are described due to inability to tolerate the diet or due to complications. These complications include metabolic derangements like dyslipidemia and hyperuricemia, gastrointestinal symptoms, renal stones, osteopenia and cardiac problems like QT prolongation and cardiomyopathy [36,59]. An uncommon yet reported complication is protein-losing enteropathy [60], and while this can be corrected for by cessation of KD, the likely consequence is an increase in seizures. Like osteoporosis and Vitamin D alterations, which may not be relevant in the setting of acute KD administration for RSE, these complications are less relevant in the setting of short-term KD. Rather, more relevant complications to be aware of include dehydration, hyponatremia, metabolic acidosis, hypoglycemia, gastroparesis and nausea/vomiting [5,59].

*Aside from poor wound healing and critical illness myopathy, our NORSE patient tolerated KD well and was discharged after 218 days in the hospital.* Additional longitudinal studies are needed to examine long-term sequelae of a high fat diet in the context of adults with SE. Future research may focus on complication rates specifically associated with acute administration of KD for adult patients with RSE/SRSE in the neurological ICU setting, as well as cessation upon symptom improvement. *Our patient’s neurological examination at discharge was significant for spontaneous eye opening, orientated x 2, minimally talkative with soft but clear speech. She was able to follow simple commands like closing her eyes and wiggle her toes. Strength was 3/5 proximal upper extremity with 2/5 in distal upper and proximal lower extremity. The patient was readmitted three weeks later for cardiac arrest, with subsequent reemergence of status epilepticus. Despite EEG improvement on restarting KD, the patient was eventually transitioned to comfort measures only.*

## 19. Future of KD in Adult SE/RSE/SRSE/NORSE

While the utility of KD in adult populations is certainly promising for the management of RSE and SRSE, there remain several gaps, including a lack of standardized treatment approach, lack of randomized, double-blind controlled studies and hidden carbohydrate sources, which may impair production of ketone bodies. These inconspicuous carbohydrates are found in commonly administered medications for SE including benzodiazepines, antibiotics, electrolyte repletion formulations and even solutions used for oral care. This review offers a brief outline of treatment strategies for KD use in adults and a systematic approach for successfully achieving, maintaining and eliminating ketosis.

## Figures and Tables

**Table 1:** Summary work-up for New-Onset Refractory Status Epilepticus (NORSE) patient.

Categories	Serum	CSF	Imaging	Pathology
Infectious:	Influenza A/B, H1N1, RPR, HIV, cat scratch panel, tick borne panel, Mycoplasma pneumonia, B Henselae, B quintana (all negative)	West Nile Virus, Enterovirus, Bacterial culture, HSV, VZV, Lyme disease, fungal culture, HHV6, EBV, Mycoplasma pneumoniae (all negative)		
Inflammatory:	ANA, dsDNA, SSA, SSB, SCL 70, CRP, ESR, TPO antibody, thyroglobulin antibody, complements (C3, C4, CH50), ANCA, B2 glycoprotein, anticardiolipin, Antiribosomal P protein Ab, ACE, smooth muscle antibody, skeletal muscle antibody	AMPA-R Ab, CASPR2 Ab, DPPX Ab, GABA-B-R Ab, GAD 65, GFAP, LGI1-IgG, mGluR1 Ab, NMDA R Ab		
Paraneoplastic:	GAD 65, NMDA, voltage gated potassium channel antibody, flow cytometry	AChR ganglionic neuronal Ab, Amphiphysin Ab, Antiglial nuclear Ab, Antineuronal nuclear Ab, CRMP-5, Neuronal (V-G) K+ channel Ab, N-Type Calcium channel ab, P/Q type calcium channel Ab, Purkinje cell cytoplasmic Ab, Striational Ab (all negative)		
Metabolic:	TSH (0.22), Free T4 (1.7 ng/dL), Ammonia (47, 33, 37 µL/dL), serum and urine toxicology (negative)			
			MRI brain w/wo contrast: restricted diffusion and hyperintense FLAIR signal in the bilateral hippocampi	Benign ovarian cyst
			CT Chest/abdomen/pelvis: no evidence of ovarian teratoma or other malignancy	No malignant cells in CSF
			US pelvis: tiny 3–4 mm echogenic focus on the left ovary which may represent a small calcification, however, a tiny teratoma cannot be excluded	
			MRI pelvis: no evidence of ovarian teratoma	

Work-up recommendations from Table 1 from Sculier C, Gaspard N. New onset refractory status epilepticus (NORSE). Seizure. 2019 May; 68:72–78. doi: 10.1016/j.seizure.2018.09.018. Epub 2018 Sep 29. PMID: 30482654. [18]. Influenza A/B: negative; H1N1: negative; Smooth and skeletal muscle antibody: negative; RPR: rapid plasma reagent—negative; ANA: antinuclear antibody—1:2560 titer; dsDNA: double-strand DNA—positive, 38.4 IU/mL; SSA: Sjögren’s Syndrome A—greater than 8; SSB: Sjögren’s Syndrome B—negative; SCL 70: Scleroderma (antitopoisomerase)—negative; Antiribosomal P protein—negative; CRP: C-reactive protein—54 mg/L; ESR: erythrocyte sedimentation rate—51 mm/h; C3/C4/CH50: within normal limits; B2 glycoprotein—negative; Anticardiolipin—negative; TPO: thyroperoxidase antibody—negative; thyroglobulin antibody—negative; ANCA: antineutrophil cytoplasmic antibody—negative; ACE: angiotensin converting enzyme—within normal limits; GAD 65: Glutamic acid decarboxylase—negative; NMDA: N-methyl-D-Aspartate receptor antibody—negative; AMPA: α-amino-3-hydroxy-5-methyl-4 -isoxazolepropionic acid receptor- antibody negative; TSH: thyroid stimulating hormone—within normal limits; HIV: Human immunodeficiency virus—negative; Lyme disease—negative; West Nile Virus—negative; Enterovirus- negative; Bacterial and fungal culture—negative; HSV: Herpes Simplex Virus—negative; VZV: Varicella Zoster Virus—negative; HHV6: Human Herpes Virus 6—negative; EBV: Epstein Barr Virus—negative; GFAP: Glial Fibrillary Acidic Protein—negative; LGI1: Leucine-rich glioma-inactivated—negative; CASPR2: Contactin-associated protein-like 2—negative; DPPX: dipeptidyl-peptidase-like protein 6—negative; GABA: gamma-aminobutyric acid—negative; mGlu1: metabotropic glutamate receptor 1—negative; CRMP-5: CV2/collapsin response mediator protein—negative; Voltage gated potassium channel—negative.

**Table 2:** Common antiseizure medications, medications utilized in hospitalized patients and associated carbohydrate, fat, and alcohol content.

**Intravenous Product (General Product Concentration)**	**Carbohydrate Excipient and Amount Per Vial**	**Carbohydrate Content at a Common Dose**	**Fat Content**	**Alcohol Content**
Brivaracetam (10 mg/mL)	–	–	–	–
Diazepam (5 mg/mL) [41]	Propylene glycol: 414 mg	828 mg CHO/10 mg	–	79 mg
Famotidine (10 mg/mL) [41]	Mannitol: 20 mg	40 mg CHO/40 mg	–	–
Fosphenytoin [42]	–	–	–	–
Lorazepam (2 mg/mL) [42]	Propylene glycol: 753 mg	–	–	–
Pentobarbital (50 mg/mL) [41]	Propylene glycol: 414 mg	–	–	79 mg
Phenobarbital (130 mg/mL) [41]	Propylene glycol: 702 mg	–	–	79 mg
Phenytoin (50 mg/mL) [41]	Propylene glycol: 414 mg	–	–	79 mg
Propofol (10 mg/mL) [41]	Glycerol: 22.5 mg/mL	450 mg CHO/h (20 mL/h)	Soybean oil: 100 mg/mL	Benzyl alcohol *
			Egg Lecithin: 12 mg/mL	
			Lipid: 100 mg/mL (1.1 kcal/mL)	
Ketamine (multiple)	–	–	–	–
Lacosamide (multiple)	–	–	–	–
Midazolam (multiple)	–	–	–	Benzyl alcohol ^†^
Thiopental (25 mg/mL)	–	–	–	–
Valproate (20 mg/mL)	–	–	–	–
Trimethoprim-sulfamethoxazole (Bactrim^®^) diluted in Dextrose 5% W 100 mL per 80–400 mg TMP-SMX	Dextrose: 5 g/100 ml	Up to 20 g CHO per dose	–	–
Vancomycin (Vancocin^®^) diluted in Dextrose 5% W per 1 g/250 mL solution	Dextrose: 5 g/100 ml	Up to 2 g CHO per dose	–	–
**Enteral Product ^§^ (General Product Strength)**	**Carbohydrate Excipient and Amount Per Unit**	**Carbohydrate Content at a Common Dose**	**Fat Content**	**Alcohol Content**
Carbamazepine (extended-release tablet)	Lactose monohydrate ^‡^ Microcrystalline cellulose ^‡^	–	–	–
Clobazam (10 mg tablet) [43]	105.3 mg/tablet	≈100 mg/10 mg	–	–
Clonazepam (0.5 mg tablet) [43]	143.5 mg/tablet	≈2800 mg/10 mg	–	–
Levetiracetam (immediate release tablet)	Croscarmellose sodium ^‡^ Polyethylene glycol 3350 ^‡^ Polyethylene glycol 6000 ^‡^	–	–	Polyvinyl alcohol
Psyllium (Metamucil) packet	9 g CHO/tablespoon	27 g CHO/day (TID)	–	–
Divalproex sodium (extended-release tablet)	Hypromelloses ^‡^ Lactose monohydrate ^‡^ Polyethylene glycol ^‡^ Propylene glycol ^‡^ Macrogol ^‡^ Microcrystalline cellulose ^‡^	–	–	*n-*Butyl alcohol Isopropyl alcohol Polyvinyl alcohol

See Appendix A for package inserts. * Present at 1.5 mg/mL in all vial sizes (0.15% *w/v*). Avoid use in pediatric populations due to benzyl alcohol content. ^†^ Present at 10 mg/mL in all vial sizes (1% *w/v*), except preservative-free formulations. Avoid use in pediatric populations due to benzyl alcohol content. ^‡^ Unknown amount of relative excipient may affect ketosis but is likely clinically insignificant. ^§^ Solution and suspension formulations should be avoided if possible as they usually contain sugars that will affect ketosis.

**Table 3:** Ketogenic Diet checklist for Status Epilepticus.

Pearls to Consider for Starting and Maintaining a Ketogenic Diet (KD)
I. KD initiation
○ Check fasting lipid panel, complete metabolic panel, complete blood count, amylase, lipase, Vitamin D serum levels
○ Record baseline weight and height
○ Continuous video EEG
○ Dietitian/nutrition consult (consider pediatric nutritionist)
○ Stop current enteral formula
○ Reduce carbohydrate content in medications and parenteral fluids with pharmacy input
○ Active communication with nursing/pharmacy, EMR warnings, and signs in room are crucial to avoid medication/IV-containing carbohydrates
○ Begin KD (e.g., KetoCal/MCT oil)
○ Include multivitamin injection, Vit. D and calcium supplementation via nasogastric tube/gastric tube
○ Change any oral agents from liquid formulation to crushed tablet formulation
II. KD maintenance
○ Remove all common carbohydrate excipients in intravenous fluids, including: ○ Glycerin ○ Maltodextrin ○ Propylene glycol ○ Sugars (dextrose, fructose, glucose, lactose, sucrose, corn syrup) ○ Sugar alcohols (glycerol, mannitol, sorbitol) ○ Starches ○ **KD can be challenged via coadministration of other meds & IVs!**
III. Pitfalls to consider:
○ Contraindications: unstable metabolic derangements, hemodynamic instability, coagulopathy/bleeding diathesis, pancreatitis, liver failure, severe hyperlipidemia, ileus, pregnancy, known fatty acid oxidation disorder or pyruvate carboxylase deficiency ○ **Propofol infusions cannot be given within 24 h before starting a KD!**

Adapted & modified from Table 3 from Thakur KT, Probasco JC, Hocker SE, et al. Neurology. 2014 Feb 25; 82(8): 665–670. [4].

## Appendix A

Briviact (brivaracetam injection) [package insert]. Smyrna, G. and I.R.M. UCB.

Cerebyx (fosphenytoin sodium injection) [package insert]. New York, N. and I.R.F. Pfizer.

Propofol injectable emulsion [package insert]. Lake Forest, I. and I.R.M. Hospira.

Ketalar (ketamine hydrochloride injection) [package Insert]. Chestnut Ridge, N., P. Pharmaceutical, and R.A. 2020.

Vimpat (lacosamide injection) [package insert]. Smyrna, G. and I.R.N. UCB.

Midazolam hydrochloride injection [package insert]. Lake Forest, I. and I.R. Hospira.

Thiopental sodium injection [package insert]. Galashiels, U. and L.R.O. Kyowa Kirin.

Valproate sodium injection [package insert]. Eatontown, N. and W.-W.P.C.R.M. 2019.

Sulfamethoxazole and trimethoprim injection [package insert]. Morgantown, W. and M.I.L.R.D. 2020.

Vancomycin hydrochloride for injection [package insert]. Rockford, I. and M.I.L.R.J. 2018.

Carbatrol (carbamazepine extended-release tablet) [package insert]. Lexington, M. and I.R.N. Takeda Pharmaceuticals America.

Levetiracetam immediate-release tablet [package insert]. Chestnut Ridge, N. and P.P.R.S. 2020.

Metamucil (psyllium husk powder) [product information]. Cincinnati, O. and P.G.R.D. 2019.

Divalproex sodium extended-release tablets [package insert]. Bridgewater, N. and A.P.L.R.A. 2020.

Peridex (chlorhexidine gluconate 0.12%) [package insert]. 3M Canada Company Dental Products. 2007.
